# Working While Caregiving in 2025

**DOI:** 10.1093/geroni/igaf122.1191

**Published:** 2025-12-31

**Authors:** Alessandra Raimondi

**Affiliations:** AARP, Washington, District of Columbia, United States

## Abstract

Roughly three-fifths of family caregivers work at some point while caregiving. Fortunately, caregivers report that employers increasingly offer workplace flexibilities and policies to help balance their responsibilities at work and at home. Many states now have paid leave laws extending protections to family caregivers. The 2020 Caregiving in the US report noted that only eight states and DC had enacted paid family leave; currently, 13 states and DC have active paid family and medical leave laws, and 15 states and DC have enacted paid sick leave laws. The nature of employment significantly influences access to caregiver-friendly benefits. Just over half of working caregivers are paid hourly, while 40 percent are salaried workers. Caregivers in salaried employment are significantly more likely to have access to a range of supportive workplace benefits than hourly-wage caregivers. Benefits access also differs for caregivers working full-time compared to those working part-time. Despite increased access to caregiver-friendly benefits and policies, most working caregivers still struggle to balance work and care, often facing significant impacts on their professional and personal lives. The share of caregivers reporting work impacts, ranging from going in late or leaving early to giving up work entirely, remains unchanged from the 2020 survey. This points to persistent gaps in meeting the needs of working family caregivers. This panel will explore access to caregiver-friendly workplace benefits, disparate access by employment type, and the common work impacts reported by caregivers trying to strike a balance between care responsibilities and work.

